# Neuroprotection of Rodent and Human Retinal Ganglion Cells In Vitro/Ex Vivo by the Hybrid Small Molecule SA-2

**DOI:** 10.3390/cells11233741

**Published:** 2022-11-23

**Authors:** Jennifer H. Pham, Gretchen A. Johnson, Rajiv S. Rangan, Charles E. Amankwa, Suchismita Acharya, Dorota L. Stankowska

**Affiliations:** 1 Department of Pharmacology & Neuroscience, University of North Texas Health Science Center, Fort Worth, TX 76107, USA; 2 The North Texas Eye Research Institute, University of North Texas Health Science Center, Fort Worth, TX 76107, USA

**Keywords:** neuroprotection, glaucoma, retinal ganglion cells, oxidative stress, neurotrophic factor, apoptosis, reactive oxygen species, mitochondria, endothelin

## Abstract

The mechanisms underlying the neuroprotective effects of the hybrid antioxidant-nitric oxide donating compound SA-2 in retinal ganglion cell (RGC) degeneration models were evaluated. The in vitro trophic factor (TF) deprivation model in primary rat RGCs and ex vivo human retinal explants were used to mimic glaucomatous neurodegeneration. Cell survival was assessed after treatment with vehicle or SA-2. In separate experiments, *tert*-Butyl hydroperoxide (TBHP) and endothelin-3 (ET-3) were used in ex vivo rat retinal explants and primary rat RGCs, respectively, to induce oxidative damage. Mitochondrial and intracellular reactive oxygen species (ROS) were assessed following treatments. In the TF deprivation model, SA-2 treatment produced a significant decrease in apoptotic and dead cell counts in primary RGCs and a significant increase in RGC survival in ex vivo human retinal explants. In the oxidative stress-induced models, a significant decrease in the production of ROS was observed in the SA-2-treated group compared to the vehicle-treated group. Compound SA-2 was neuroprotective against various glaucomatous insults in the rat and human RGCs by reducing apoptosis and decreasing ROS levels. Amelioration of mitochondrial and cellular oxidative stress by SA-2 may be a potential therapeutic strategy for preventing neurodegeneration in glaucomatous RGCs.

## 1. Introduction

Glaucoma is a group of multifactorial optic neuropathies characterized by retinal ganglion cell (RGC) degeneration, axonal transport deficits, and progressive excavation of the optic nerve head [1]. Primary open-angle glaucoma (POAG) is the most common form of the disease, with elevated intraocular pressure (IOP) being a major risk factor [2]. Approximately 112 million people worldwide are predicted to have glaucoma by 2040 with over 50% being unaware of their condition [3,4,5]; it is, therefore, essential to develop therapies that can prevent glaucomatous vision loss. Current pharmacological therapies for glaucoma mainly aim to lower IOP but do not fully address the progressive loss of RGCs, which continues to occur despite IOP reduction [2].

In the central nervous system, retinal ganglion cells process and transmit visual information from the eyes to the brain. As these neurons are terminally differentiated, they cannot regenerate if injured [6]. Due to their high metabolic demand, RGCs are susceptible to oxidative damage [7]. This is, in part, due to insufficient mitochondrial energy generation by injured unmyelinated RGC axons, and the increased ATP requirements of demyelinated axons [7,8,9]. During the process of ATP production, the mitochondria generate reactive oxygen species (ROS), which become increasingly abundant when energy demand is heightened under pathological conditions [7]. This means that the mitochondria are at a higher risk of oxidative damage. This is particularly relevant to the mitochondria-rich RGCs that have a high energy demand and rely on oxidative metabolism.

Oxidative damage occurs when there is an imbalance between the production of ROS during physiological processes and the clearance of ROS by endogenous (superoxide dismutase, catalase, glutathione, etc.) and exogenous (vitamin C, vitamin E, carotenoids, etc.) antioxidants [10,11,12]. Oxidative damage also increases the release of cytochrome c from the mitochondria into the cytosol, which acts as the major initiator of the apoptotic signaling cascade [7,13]. ROS, such as superoxide anions, have been demonstrated to be involved in apoptotic signaling after axonal injury [14,15]. Another signal that can induce apoptosis in RGCs is trophic factor (TF) deprivation. Neurotrophic factors, such as brain-derived neurotrophic factor (BDNF) and ciliary neurotrophic factor (CNTF), are essential for RGC survival. The deprivation of these factors resulting from the blockage in retrograde axonal transport to the RGC soma as well as their degradation due to increased ROS levels may trigger apoptosis in RGCs [16,17]. Treatment using these neurotrophic factors have demonstrated protection of RGCs in varying experimental models of injury [18,19].

Endogenous antioxidants, such as superoxide dismutase (SOD), show a decline in the retina in pre-clinical glaucoma models such as ischemia/reperfusion (I/R) and optic nerve crush (ONC) leading to RGC death [20,21,22]. Treatment of RGCs with SOD in ONC and ischemia rodent models has been shown to increase RGC survival [21,22] and improve visual functions in other disease-induced retinal degenerations, such as diabetes [23]. We previously reported that a hybrid molecule, SA-2, containing both a nitric oxide (NO) donating group (to decrease IOP through the cGMP pathway) and a SOD mimetic effectively scavenged the ROS generated in vitro and increased SOD activity in rodent models of RGC death [24,25,26].

Compound SA-2 has been proven to be effective in protecting RGCs in the optic nerve crush model and ischemia/reperfusion model in mice. We also demonstrated that SA-2 was efficacious in lowering IOP in two rodent models of ocular hypertension [25]. Our current study evaluated the neuroprotective and antioxidant effects of SA-2 and its potential mechanisms in three models of RGC injury. Free radicals generated during glaucoma pathology deplete the NO bioavailability and contribute to the death of trabecular meshwork cells and RGCs [27,28,29,30,31]. The goal of the study was to investigate SA-2-mediated RGC protection from free radical-induced death. For this purpose, we used in vitro and ex vivo trophic factor (TF) deprivation model in primary rat RGCs and human retinal explants, ex vivo *tert*-Butyl hydroperoxide (TBHP)-induced oxidative stress model in rat retinal explants, and in vitro endothelin-3 (ET-3)-induced oxidative stress model in primary RGCs. Since axotomy of the optic nerve has been shown to induce a superoxide burst independent of the lack of neurotrophic factors, we evaluated SA-2′s ability to protect against two causes that induce RGC death via apoptosis. SA-2 demonstrated significant protection of RGCs through inhibition of apoptosis and scavenging of reactive oxygen species.

## 2. Materials and Methods

### 2.1. Compound SA-2

Compound SA-2 was synthesized following the previously published protocol developed by our lab [24], and purified using liquid chromatography and recrystallization. The structure of SA-2 was characterized using ^1^HNMR and mass spectroscopy. SA-2 was reconstituted in Dulbecco’s phosphate-buffered saline (DPBS) and diluted into the cell culture medium.

### 2.2. Animal Care and Protocol Approval

All procedures involving animals were carried out following the ARVO resolution for the Use of Animals in Ophthalmic and Vision Research and approved by the University of North Texas Health Science Center (UNTHSC) Institutional Animal Care and Use Committee (IACUC 2019-0036). Sprague Dawley rat pups (post-natal days 4 to 7) were used for the isolation of primary retinal ganglion cells. The pups were born from pregnant dams purchased from Charles River Laboratories (Wilmington, MA, USA). The animals were housed under bright light conditions with food and water provided ad libitum. The adult (9–13 months) Sprague Dawley females were used for the rat retinal explants experiments.

### 2.3. Trophic Factor Deprivation Model in Primary RGCs

**Figure 1:**
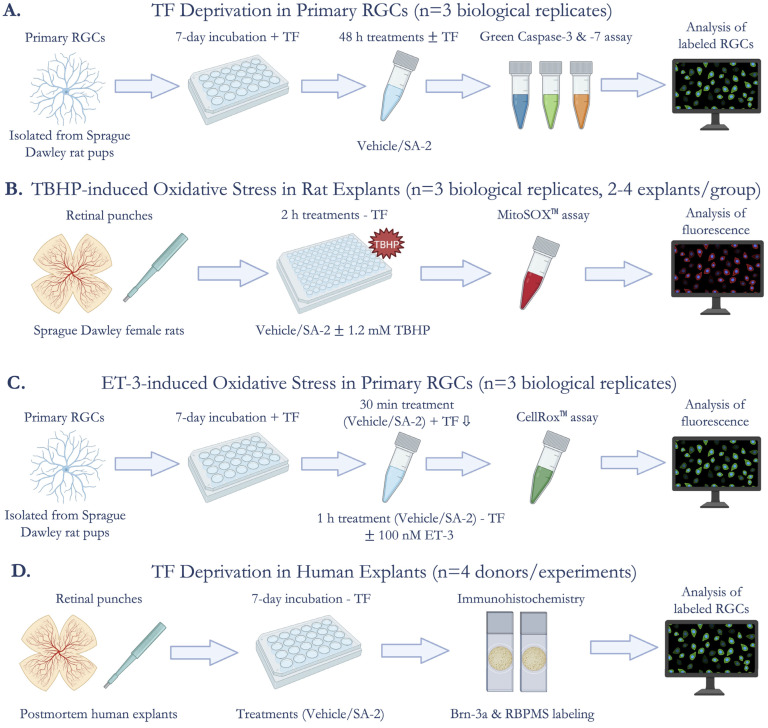
Experimental Design. (**A**) Cell survival assessment after trophic factor (TF) deprivation in primary rat retinal ganglion cells (RGCs) (n = 3 biological replicates) using Image-iT™ LIVE Green Caspase-3 and -7 Detection Kit; (**B**) Measurement of mitochondrial superoxides produced by *tert*-Butyl hydroperoxide (TBHP)-induced oxidative stress in ex vivo rat retinal explants (n = 3 biological replicates, 2–4 explants per group) using MitoSOX™ Red Mitochondrial Superoxide Indicator; (**C**) Quantification of reactive oxygen species (ROS) in endothelin-3 (ET-3)-induced oxidative stress in primary rat RGCs (n = 3 biological replicates) using CellROX™ Green Reagent; (**D**) Assessment of cell survival after TF deprivation in ex vivo human retinal explants (n = 4 donors/experiments) by immunostaining for Brn-3a and RBPMS (RGC markers).

Primary cultures of rat retinal ganglion cells were isolated according to the previously published two-step panning method [32] from Sprague Dawley rat pups (post-natal days 4 to 7) (n = 3 biological replicates). Approximately 35,000 primary RGCs were seeded on glass coverslips coated with poly-D-lysine (Sigma-Aldrich Corp., St. Louis, MO, USA) and mouse laminin (Trevigen Inc., Gaithersburg, MD, USA), and cultured in Dulbecco’s Modified Eagle Medium (DMEM; Thermo Fisher Scientific, Waltham, MA, USA) containing brain-derived neurotrophic factor (BDNF; 50 ng/mL; Peprotech, Rocky Hill, NJ, USA), ciliary neurotrophic factor (CNTF; 10 ng/mL; Peprotech), and forskolin (5 ng/mL; Sigma-Aldrich Corp., St. Louis, MO, USA). The cells were incubated at 37 °C in 10% CO_2_ and 90% air. One-half of the culture medium was changed every two days. After incubating in culture with neurotrophic factors for one week to promote neurite outgrowth, the RGCs were treated with either vehicle (DPBS) or SA-2 [0.1 mM, 0.5 mM, or 1 mM] in the presence or absence of neurotrophic factors for 48 h. Image- iT™ LIVE Green Caspase-3 and -7 Detection Kit (I35106, Invitrogen, Carlsbad, CA, USA) was used to stain apoptotic and dead RGCs according to the manufacturer’s instructions. Primary RGCs were imaged by Cytation 5 (Agilent, Santa Clara, CA, USA). The labeled RGCs were manually counted in a masked manner using ImageJ/Fiji Software. Cell survival was expressed as a ratio of total treated cells to total cells in the vehicle control group (Figure 1A).

### 2.4. TBHP-Induced Oxidative Stress Model in Ex Vivo Rat Retinal Explants

Adult rat retinal explant cultures were prepared as described previously by our lab group [33] from Sprague Dawley female rats (9–13 months). Explants isolated from each retina were incubated in a 96-well plate in Hank’s Balanced Salt Solution (HBSS; Thermo Fisher Scientific, Waltham, MA, USA) (n = 3 biological replicates, 2–4 explants/treatment group). The explants were treated with either vehicle (DPBS) or SA-2 [1 mM] in the presence or absence of *tert*-Butyl hydroperoxide (TBHP, [1.2 mM]) for 2 h at 37 °C in 5% CO_2_ and 95% air. The MitoSOX™ Red Mitochondrial Superoxide Indicator (M36008, Invitrogen, Carlsbad, CA, USA) was used to stain for superoxides produced in the mitochondria according to the manufacturer’s instructions. Fluorescence was captured using Cytation 5 (Agilent, Santa Clara, CA, USA). The integrated density from four regions of interest on each image was measured using ImageJ/Fiji. The average integrated density was calculated and plotted (Figure 1B).

### 2.5. Endothelin-3-Induced Oxidative Stress Model in Primary RGCs

Primary cultures of rat RGCs were prepared as described above. After incubating for 7 days with neurotrophic factors, the RGCs were pre-treated with either vehicle (DPBS) or SA-2 [0.1 mM, 0.5 mM, or 1mM] for 30 min, and incubated for 1 h in the presence or absence of 100 nM of endothelin-3 (ET-3) (4013291.0500, BACHEM, Torrance, CA, USA). CellROX™ Green Reagent for oxidative stress detection (C10444, Invitrogen, Carlsbad, CA, USA) was used to stain reactive oxygen species in the cells according to the manufacturer’s instructions. Fluorescence was captured using Cytation 5 (Agilent, Santa Clara, CA, USA) and the integrated density was measured using ImageJ/Fiji (Figure 1C).

### 2.6. Trophic Factor Deprivation Model in Ex Vivo Human Retinal Explants

Human retinas were isolated from healthy donors within 12 h postmortem and were free of any diagnosed retinal pathology or neurodegenerative conditions that could have affected the condition of the retinas (n = 4 donors/experiments). Four to seven explants were isolated from each retina and incubated with the RGC layer facing up on Transwell Permeable 6.5 mm inserts. Four explants from each experiment were fixed with 4% paraformaldehyde at 4 °C for 24 h following axotomy and were used as the control group (0 DEV). The rest of the explants were cultured for 7 days (7 DEV) following axotomy without neurotrophic factors and treated either with the vehicle (DPBS) or SA-2 [1 mM] in an explant medium consisting of phenol red-free Neurobasal A with 2% B-27, 1% N-2, 0.8 mM L-glutamine, 100 U/mL penicillin, and 100 μg/mL streptomycin (all reagents were purchased from Thermo Fisher Scientific, Waltham, MA, USA) using the modified protocol published by Osborne et al., 2018 [34]. Following the 7-day treatments, the explants were fixed with 4% paraformaldehyde at 4 °C for 24 h for further analysis. All explants were permeabilized with the permeabilization buffer (0.1% sodium citrate, 0.2% Triton X-100 in 1X PBS), blocked with blocking buffer (5% normal donkey serum, 5% BSA in 1X PBS), and incubated with primary antibodies: goat anti-Brn-3a antibody (sc-31984, dilution 1:250; Santa Cruz Biotechnology, Dallas, TX, USA) [35] and rabbit anti-RBPMS antibody (GTX118619, dilution 1:250; GeneTex, Irvine, CA, USA) [36] at 4 °C for 72 h. Then, fluorescently labeled secondary antibodies: donkey anti-goat Alexa 488 and donkey anti-rabbit Alexa 647 (A11055, A31573, both dilution 1:1000; Invitrogen, Carlsbad, CA, USA) were added, incubated for 2 h at 4 °C, and retinal explants were mounted with FluorSave reagent (345789, Sigma-Aldrich Corp., St. Louis, MO, USA) on glass slides. The explants were imaged using Leica DMi8 Confocal Microscope and labeled RGCs were counted in a masked, semi-automatic manner using the cell counter plugin (http://imagej.nih.gov/ij/; provided in the public domain by the National Institutes of Health, Bethesda, MD, USA, accessed on 7 July 2022) (Figure 1D).

### 2.7. Statistical Analysis

Statistical analysis was performed using GraphPad Prism version 9.4.1 for Mac OS, GraphPad Software, San Diego, California USA, www.graphpad.com, accessed on 18 July 2022. Kolmogorov–Smirnov test for normality was performed and the nonparametric Kruskal–Wallis test followed by Dunn’s multiple comparison test, or Mann–Whitney test was used. All data presented are the mean ± standard error of the mean (SEM) from at least 3 biological replicates and 2 technical replicates per treatment group. Values of *p* < 0.05 were considered statistically significant. * *p* < 0.05, ** *p* < 0.01, *** *p* < 0.001, **** *p* < 0.0001.

## 3. Results

### 3.1. SA-2 Is Neuroprotective to Primary RGCs in the Trophic Factor Deprivation Model

Neurotrophic factors are necessary for the survival of RGCs. In this study, we used the deprivation of these essential trophic factors as a model of RGC death. The primary RGCs were treated with SA-2 at three concentrations [0.1 mM, 0.5 mM, and 1 mM] in the presence or absence of neurotrophic factors for 48 h. The apoptotic and dead cells were stained using the Image-iT™ LIVE Green Caspase-3 and -7 Detection Kit and the number of RGCs were counted.

**Figure 2:**
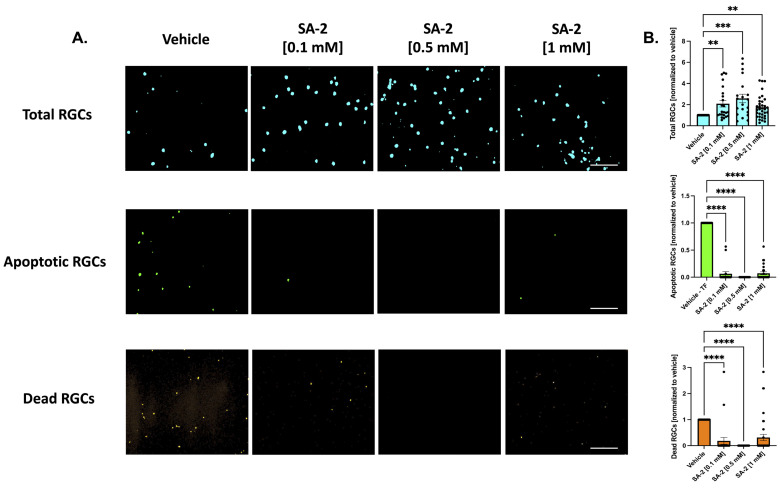
Treatment of primary retinal ganglion cells (RGCs) from Sprague Dawley rat pups in the absence of neurotrophic factors. (**A**) Representative images of RGCs treated with either vehicle (Dulbecco’s phosphate-buffered saline/DPBS) or SA-2 in the absence of neurotrophic factors for 48 h; (**B**) Graph bars represent total (blue, Hoechst 33342), apoptotic (green, active caspase-3 and caspase-7), and dead (orange, propidium iodide) RGC counts as a ratio to the total cells in the vehicle control group. In the absence of neurotrophic factors, SA-2 mediated a significant decrease in apoptotic cells by 92.78% (*p* < 0.0001) and a 68.57% (*p* < 0.0001) decrease in dead cells, using Kruskal–Wallis followed by Dunn’s multiple comparisons. Data represent the mean ± standard error of the mean (SEM) (n = 3 biological replicates). ** *p* < 0.01, *** *p* < 0.001, **** *p* < 0.0001. The scale bar represents 200 µm.

As expected, TF deprivation caused a loss in RGCs by an average of 37.83% as demonstrated in Figure S1 (*p* < 0.001). Treatments with SA-2 significantly improved RGC survival following TF deprivation (Figure 2A, teal color) at all concentrations (0.1 mM: 108.87% increased preservation, *p* < 0.01; 0.5 mM: 158.93%, *p* < 0.001; 1 mM: 74.96%, *p* < 0.01) compared to the vehicle (DPBS)-treated group (Figure 2B, teal color). The number of apoptotic cells (Figure 2A, green color) significantly decreased following SA-2 treatment, compared to the vehicle control at 0.1 mM (93.72% decrease, *p* < 0.0001), 0.5 mM (99.99%, *p* < 0.0001), and 1 mM (92.78%, *p* < 0.0001) (Figure 2B, green color). Finally, the number of dead RGCs (Figure 2A, orange color) significantly decreased with SA-2 treatment at each concentration (0.1 mM: 81.67% decrease, *p* < 0.0001; 0.5 mM: 99.99%, *p* < 0.0001; 1 mM: 68.57%, *p* < 0.0001) (Figure 2B, orange color).

**Figure 3:**
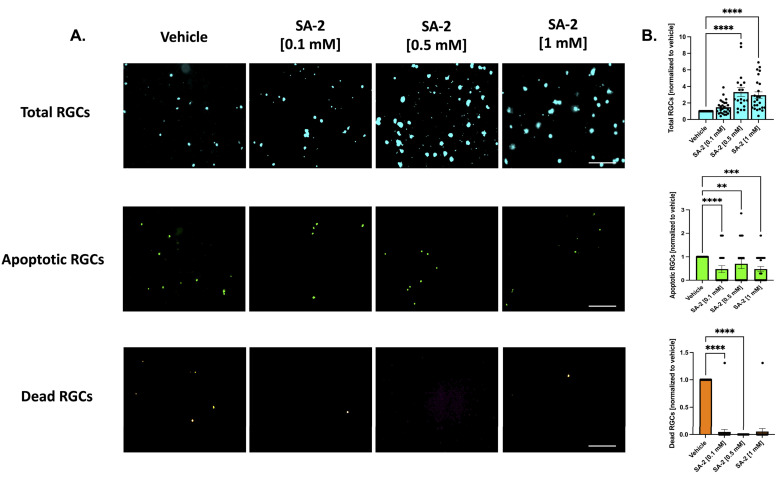
Treatment of primary RGCs from Sprague Dawley rat pups in the presence of neurotrophic factors. (**A**) Representative images of RGCs treated either with vehicle (DPBS) or SA-2 in the presence of neurotrophic factors for 48 h. (**B**) Graph bars represent total (blue, Hoechst 33342), apoptotic (green, active caspase-3 and caspase-7), and dead (orange, propidium iodide) RGC counts as a ratio to the total cells in the vehicle control group. In the presence of neurotrophic factors, SA-2 mediated a significant decrease in apoptotic cells by 30% (*p* < 0.01) and a 83.65% (*p* < 0.01) decrease in dead cells, using Kruskal–Wallis followed by Dunn’s multiple comparisons. Data represent the mean ± SEM (n = 3 biological replicates). ** *p* < 0.01, *** *p* < 0.001, **** *p* < 0.0001. The scale bar represents 200 μm.

Similarly, in the presence of neurotrophic factors, SA-2 significantly preserved the total number of RGCs (Figure 3A, in teal) at 0.5 mM (232.45% increase, *p* < 0.0001) and 1 mM (194.74%, *p* < 0.0001) (Figure 3B, in teal). Even in the presence of neurotrophic factors, the primary RGCs are naturally dying off. However, the number of apoptotic cells (Figure 3A, in green) significantly decreased in the presence of SA-2 at each concentration (0.1 mM: 52.5% decrease, *p* < 0.0001; 0.5 mM: 30%, *p* < 0.01; 1 mM: 52.48%, *p* < 0.001) (Figure 3B, in green). The number of dead RGCs (Figure 3A, in orange) also significantly decreased after SA-2 treatment at 0.1 mM (95.33% decrease, *p* < 0.0001) and 0.5 mM (99.99%, *p* < 0.0001) compared to the vehicle control (Figure 3B, in orange). These data suggested that SA-2 protected the RGCs from apoptotic cell death in the TF deprivation model.

### 3.2. SA-2 Scavenges Reactive Oxygen Species in TBHP-Induced Oxidative Stress Model in Ex Vivo Rat Retinal Explant

**Figure 4:**
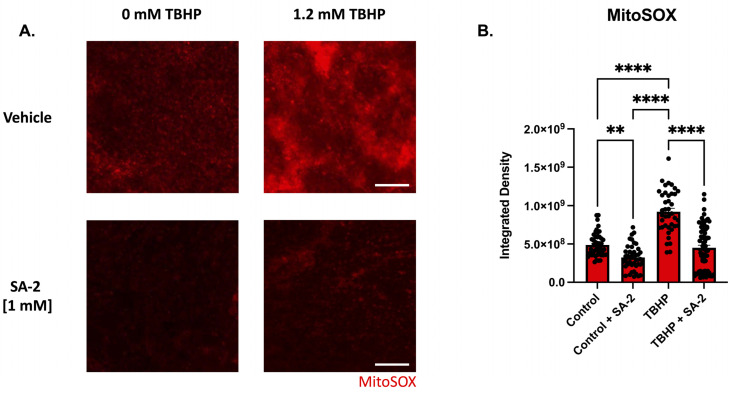
TBHP-mediated oxidative stress in retinal explants from adult Sprague Dawley rats. (**A**) Representative images of superoxide formation stained with MitoSOX™ following 2 h of treatment with control [0 mM] and [1.2 mM] TBHP in the presence of either vehicle (DPBS) or SA-2 [1 mM]. The scale bar represents 100 µm. (**B**) Graph bars demonstrate a decrease in mitochondrial stress amount quantified by MitoSOX™ integrated density in the SA-2 treated groups compared to the vehicle-treated groups (control (0 mM TBHP): 33.74% decrease, *p* < 0.01; TBHP (1.2 mM): 51.1%, *p* < 0.0001) using Kruskal–Wallis followed by Dunn’s multiple comparisons. Data represent the mean ± SEM (n = 3 biological replicates, 2–4 explants/group). ** *p* < 0.01, **** p < 0.0001.

To evaluate SA-2′s ability to prevent oxidative damage in ex vivo rat retinal explants, we induced oxidative stress by treatment with 1.2 mM *tert*-Butyl hydroperoxide (TBHP) (determined by dose–response study). The retinal explants were incubated with either vehicle (DPBS) or SA-2 [1 mM] for 2 h in the presence or absence of TBHP. Since the mitochondria are a major source of ROS production and are particularly susceptible to oxidative damage, superoxides produced by the mitochondria were stained using the MitoSOX™ red mitochondrial superoxide probe and the measured integrated density was analyzed (Figure 4). In the presence of TBHP, the measured integrated density was significantly increased by 88.77% (*p* < 0.0001) indicating an increase in the number of mitochondrial superoxides produced in the cells compared to the control group (Figure 4B). However, with SA-2 [1 mM] treatment, integrated density was significantly decreased by 51.1% (*p* < 0.0001) compared to the TBHP group. Additionally, there was a significant decrease in the integrated density following SA-2 treatment by 33.74% (*p* < 0.01) when compared to the vehicle-treated group, suggesting that SA-2 is effective in reducing the number of superoxides produced by the mitochondria even in the absence of TBHP (0 mM TBHP, Figure 4B).

### 3.3. SA-2 Prevents Reactive Oxygen Species Formation in ET-3-Induced Oxidative Stress in Primary RGCs

**Figure 5:**
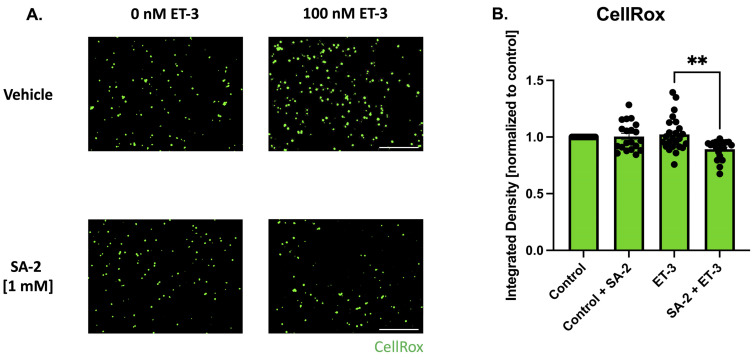
Endothelin-3-mediated oxidative stress in primary RGCs isolated from Sprague Dawley rat pups. (**A**) Representative images of ROS formation stained with CellROX™ Green following 1 h of treatment with control [0 nM] and [100 nM] ET-3 in the presence of either vehicle (DPBS) or SA-2 [1 mM]. The scale bar represents 1000 µm. (**B**) Bar graph demonstrates a decrease in ROS amount quantified by CellROX™ Green integrated density in the SA-2 treated group compared to the vehicle-treated group in the presence of ET-3 (13.01% decrease, *p* < 0.01) using Kruskal–Wallis followed by Dunn’s multiple comparisons. Data represent the mean ± SEM (n = 3 biological replicates). ** *p* < 0.01.

Upregulation of endothelin and its receptors (endothelin A and B) has been implicated in the pathology of glaucoma [37,38,39]. Elevated levels of ET-1 were observed in glaucoma patients [39] which can lead to decreased ocular blood flow and activate signaling pathways, including the JNK pathway, which in turn contribute to the degeneration of retinal ganglion cells [37,38,40]. In this study, endothelin-3 was used to induce oxidative stress in primary RGCs isolated from Sprague Dawley rat pups. The cells were pre-treated with SA-2 [1 mM] for 30 min before being co-treated in the presence or absence of ET-3 [100 nM] for 1 h. ROS produced by the cells were stained using the CellROX™ Green probe and the measured integrated density was analyzed (Figure 5). In the SA-2 + ET-3 treated group, the measured integrated density decreased by 12.72% (*p* < 0.01) (Figure 5B), indicating that SA-2 was able to scavenge the ROS generated in the cells.

### 3.4. SA-2 Protects RGCs in Ex Vivo Human Retinal Explant Trophic Factor Deprivation Model

**Figure 6:**
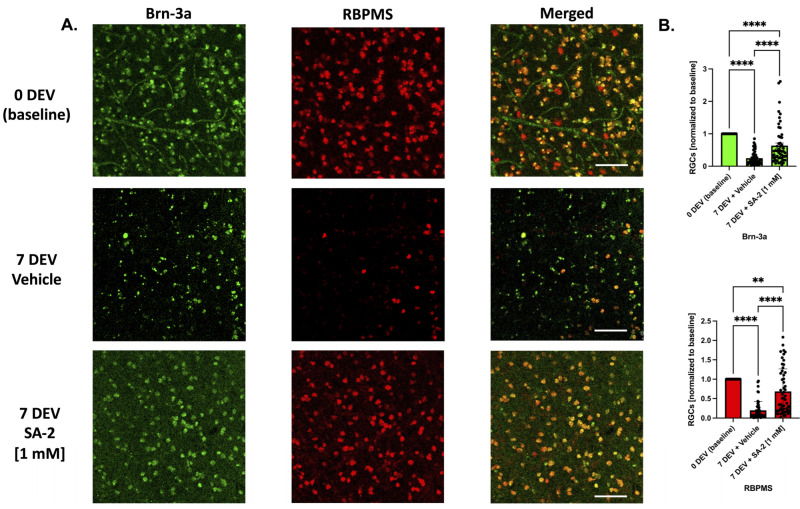
Ex vivo trophic factor deprivation model in human explants. (**A**) Representative images of the human RGCs at 0-day ex vivo (DEV), 7-day ex vivo (7 DEV) with the vehicle (DPBS), and 7 DEV with SA-2 [1 mM]. RGCs were stained with RGC markers Brn-3a (green) and RBPMS (red). The scale bar represents 100 µm. (**B**) RGC count showed preservation of RGCs treated with SA-2 at 7 DEV compared to the vehicle-treated group in both Brn-3a (156.13% increase, *p* < 0.0001) and RBPMS staining (249.38%, *p* < 0.001) using Kruskal–Wallis followed by Dunn’s multiple comparisons. Data represent the mean ± SEM (n = 4 donors/experiments). ** *p* < 0.01, **** *p* < 0.0001.

To test the translational potential of SA-2 treatment to protect human RGCs, we used the TF deprivation model in ex vivo postmortem axotomized human retinal explants. The explants were incubated for 7 days ex vivo (7 DEV) with either vehicle (DPBS) or SA-2 [1 mM] in the absence of neurotrophic factors. Then, the explants were immunostained with RGC-specific markers Brn-3a (in green) and RBPMS (in red) and cell survival was assessed (Figure 6A). At 7 days following axotomy, there was a significant decrease of RGC numbers in both vehicle-treated (Brn-3a: 75.2% decrease, *p* < 0.0001; RBPMS: 80.41%, *p* < 0.0001) and SA-2 treated groups (Brn-3a: 36.48% decrease, *p* < 0.0001; RBPMS: 31.57%, *p* < 0.01) compared to the 0-day ex vivo (0 DEV) baseline group (Figure 6A,B). However, there was a significant preservation of RGCs in the SA-2 treated group compared to the vehicle-treated group in Brn-3a labeling (156.13% increased preservation, *p* < 0.0001) and RBPMS immunostaining (249.38%, *p* < 0.0001) (Figure 6B). This suggests that SA-2 was effective at protecting human RGCs undergoing glaucomatous insult and cell death, with potential future use for glaucoma patients.

## 4. Discussion

The approach of using a single compound that is metabolized into two active molecules is a relatively new concept in the treatment of POAG. The only FDA-approved drug formulated in this manner is Latanoprostene Bunod (LBN, Vyzulta™), which is a prodrug composed of a prostaglandin F_2_α analog with an attached NO-donating moiety [41]. LBN is administered topically and metabolized in the anterior chamber, where its metabolite latanoprost acid induces extracellular matrix rearrangements to promote increased uveoscleral aqueous humor outflow and the released NO induces cytoskeletal changes that promote increased outflow of aqueous humor through the trabecular meshwork [41,42,43]. As such, the dual action of LBN is focused on enhancing the drug’s IOP-lowering effect. Although IOP reduction does slow glaucomatous neurodegeneration, these mechanisms of action do not directly intervene in the pathophysiological processes within the retina.

SA-2, the small hybrid molecule developed in our lab, has previously been evaluated as a potential new approach for the treatment of glaucoma in two rodent models of RGC death and two rodent models of ocular hypertension [24,25,26]. Here, we utilized a variety of injury models to evaluate possible mechanisms of action of SA-2 in the protection of RGCs. Specifically, we utilized three models of RGC death: TF deprivation, TBHP-induced oxidative stress, and ET-3-induced oxidative stress. The TF deprivation model allowed us to evaluate whether SA-2 was able to interfere in the apoptotic signaling pathways initiated by TF deprivation [16]. SA-2 treatment in the absence of neurotrophic factors significantly decreased the number of apoptotic RGCs, as determined by staining for active caspase-3 and -7, indicating a potential mechanism that deterred cell death activation by these two caspases. With superoxide anions being implicated in apoptotic signaling, we also wanted to assess whether SA-2 protected the RGC mitochondria from oxidative damage. Elevated levels of ET-1 were observed in glaucoma patients [39] which can lead to decreased ocular blood flow and activate signaling pathways, including the JNK pathway, which in turn contribute to the degeneration of retinal ganglion cells [37,38,40]. Primary RGCs treated with the ET_B_ receptor agonist, ET-3, demonstrated almost complete withdrawal of neurites and also showed significantly higher number of EtHD staining, indicative of cell death [44].

Our work demonstrated that SA-2 significantly scavenged mitochondrial superoxides in the rat retinal explants and ROS in primary RGCs following TBHP- and ET-3-induced oxidative stress, respectively. This indicates SA-2′s involvement in inhibiting two significant mechanisms of RGC apoptosis: oxidative damage and TF deprivation. Although the concentrations of SA-2 used in this study are higher than the desired clinical therapeutic dose, none of the doses of SA-2 used showed any toxicity to primary RGCs in the presence or absence of neurotrophic factors. Further experimentation will be performed to test the neuroprotective potential of SA-2 in RGCs at lower doses.

There are few preclinical models available for assessing the effect of new therapeutic agents in human RGCs. Primary RGCs are challenging to culture, and limited success has only been achieved with cells derived from embryonic tissues [45]. Alternative methods such as the use of induced human pluripotent stem cells (iPSCs) have become more viable recently but are still limited in their translatability to true RGCs as they exist within the retinal environment [46,47]. Human retinal tissue explants, however, serve as an excellent way of evaluating the translational potential of new drugs, such as SA-2, by allowing us to assess the effect of these drugs on a heterogeneous population of RGCs within the physiologic context of a live retinal section [34,48].

During glaucomatous neurodegeneration, increased oxidative stress is believed to contribute to RGC loss [7,49]. Numerous in vivo studies have demonstrated the potential of antioxidant administration in protecting against RGC death in both hypertensive and pressure-independent models of glaucoma [50,51,52]. For instance, the antioxidant 4-hydroxy TEMPOL, a SOD mimetic, decreased retinal inflammation in rodent ocular hypertensive models, even in SOD knockout mice—which exhibited a disproportionately strong inflammatory response in the retina after induction of ocular hypertension [53,54]. This demonstrates how compounds like TEMPOL may protect against oxidative stress-induced damage in neural tissues by restoring antioxidant defense systems that become defective during various pathophysiological processes [53,55].

Compound SA-2 is a structural analog of 4-hydroxy TEMPOL and is designed to be more potent and multifunctional. SA-2 utilizes a modern “dual-action” design like the commercially available LBN but takes advantage of emerging evidence for the use of antioxidants in the treatment of glaucoma through its TEMPOL-like activity. Previously we reported that in ex vivo rat retinal explants, SA-2 demonstrated protection of RGCs against hypoxic damage at a lower concentration of 1 μM [26] whereas 4-hydroxy TEMPOL was efficacious at a very high concentration of 5 mM in primary RGCs in a model of TNF-α and hypoxia-induced RGC damage [54]. Additionally, SA-2 treatment was shown to upregulate SOD activity in the mouse retinas following ONC [26], suggesting a potential mechanism of neuroprotective action of SA-2 in the retinas of mice subjected to ONC. In the current study, SA-2 prevented an increase in active caspase-3 and -7 levels following TF deprivation in axotomized human and rodent RGCs likely through SOD mimetic activity as well. We also reported that SA-2 was effective at protecting the RGCs at doses ranging from 75 pM (measured concentration in retinal tissue after 2% SA-2 dosing) in vivo to 1 μM concentration ex vivo in three acute models of RGC death that also cause oxidative damage: hypoxia, optic nerve crush, and ischemia/reperfusion model [25,26], and is more efficacious than 4-hydroxy TEMPOL at 5 mM. Thus, SA-2 has potential as a novel glaucoma therapeutic by both lowering IOP and protecting against oxidative damage in sensitive ocular tissues, such as the trabecular meshwork and retina [31].

While it is evidenced that SA-2′s ROS scavenging moiety significantly contributed to the preservation of RGCs, the potential neuroprotective role of the NO-donating moiety should also be investigated. The effect of NO has been observed to have dichotomous regulatory roles. These effects could be modulated by direct or indirect interactions and exert differential dose-dependent or cell-specific activities [56]. Studies have shown nitric oxide can be detrimental to RGCs at higher concentrations but beneficial at lower concentrations, depending on the NO donor [42,57,58,59]. S-nitroso-N-acetylpenicillamine (SNAP), an NO releaser, significantly reduced the number of RGCs at concentrations of 100 μM or higher but was neuroprotective at 41 μM or lower [57]. SNAP also demonstrated inhibition of apoptosis in serum-deprived cortical neurons, while its high dose acted as a neurotoxic agent in the serum-supplemented medium. Under serum deprivation, SNAP inhibited caspase-3 activation and/or increased antiapoptotic protein levels (Bcl-2/Bcl-x(L)) [59]. It has also been shown in other studies that inhibition of apoptosis by NO is at the level of caspases and partly mediated by S-nitrosylation [60,61].

The novelty of incorporating the piperidine nitroxide (TEMPOL) moiety into the sydnonimine core of SA-2 facilitates robust NO release by scavenging both ROS and the resulting reactive nitrogen species such as peroxynitrite (ONOO^.-^) (Figure S2) [24]. This provides an opportunity for NO to work while preventing deleterious effects from its by-products [62]. At 1 mM concentration, SA-2 still demonstrated higher RGC preservation compared to the control in the TF deprivation model in primary culture and human explants.

The field of nanomedicine has advanced tremendously in recent years, starting with the implementation of nanotherapy in cancer treatments [63,64]. The nanodelivery method can potentially distribute SA-2 effectively to the RGCs while limiting side effects due to off-target delivery or higher drug concentrations [65,66]. SA-2-loaded polymeric nanoparticles (SA-2-NPs), through topical administration, have demonstrated availability in the back of rat eyes with the highest concentration found in the retina (9.7 pg/mg) [25]. For future experiments, further characterization of SA-2-NPs in RGCs will be evaluated through cytotoxicity and cell uptake assays. The nanoparticles will also be evaluated in our oxidative damage and TF deprivation models to compare the efficacy to pure SA-2.

In summary, SA-2 demonstrated neuroprotection in primary RGCs isolated from rat pups, in rat retinal explants, and human explants by blocking apoptosis and decreasing ROS levels in the TF deprivation model as well as the TBHP- and ET-3-induced oxidative stress models. Compound SA-2 was found to be safe in the retina and scavenged ROS produced by the mitochondria and other cellular organelles. These findings support SA-2′s potential for protection of the RGCs against oxidative damage seen in optic neuropathies such as glaucoma and other neurodegenerative diseases where oxidative stress and apoptotic cell death are involved. Further investigation to analyze the detailed mechanism(s) of action of SA-2 and modulation of pathways implicated in glaucoma is ongoing. Our results indicate that the neuroprotective action of compound SA-2 is mediated through its antioxidant and mitoprotective activity in RGCs. Furthermore, SA-2 demonstrated neuroprotection in human retinal explants thus increasing the clinical translatability of this compound for the treatment of glaucomatous optic neuropathy.

## Data Availability

The data presented in this study are available on request from the corresponding author.

## Supplementary Materials

The following supporting information can be downloaded at: https://www.mdpi.com/article/10.3390/cells11233741/s1, Figure S1: Vehicle RGC counts in the presence and absence of trophic factors. Figure S2: The structure of SA-2 and 4-hydroxy TEMPOL.

## Abbreviations

4-hydroxy	TEMPOL/TEMPOL 4-hydroxy-2,2,6,6-tetramethyl piperidinyl-1-oxyl
BDNF	Brain-derived Neurotrophic Factor
CNTF	Ciliary Neurotrophic Factor
ET-3	Endothelin-3
IOP	Intraocular Pressure
I/R	Ischemia/Reperfusion
NO	Nitric Oxide
POAG	Primary Open Angle Glaucoma
ROS	Reactive Oxygen Species
RGC	Retinal Ganglion Cell
SOD	Superoxide Dismutase
TBHP	*tert*-Butyl Hydroperoxide
TF	Trophic Factor
