# Supplementary material for: Neuroprotection of Rodent and Human Retinal Ganglion Cells In Vitro/Ex Vivo by the Hybrid Small Molecule SA-2
Source: Cells. 2022 Nov 23;11(23):3741. doi: 10.3390/cells11233741 (PMC9735605; doi:10.3390/cells11233741)
Supplement: Supplementary file 1 [file cells-11-03741-s001.zip › cells-2005948-supplementary.pdf]

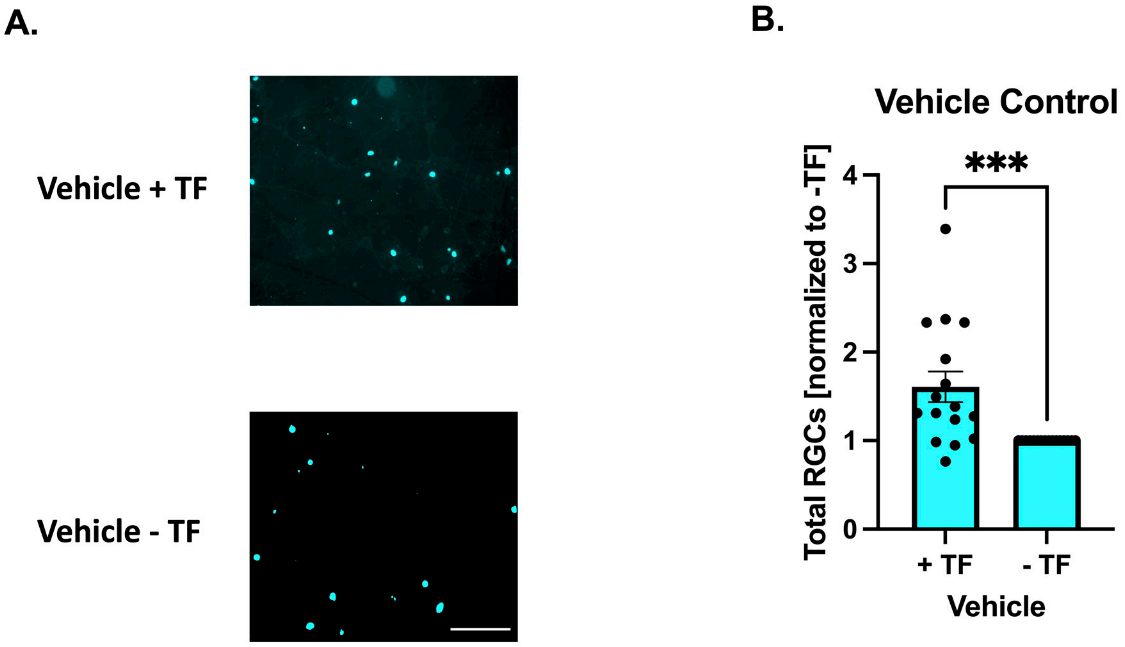

Figure S1: Vehicle RGC counts in the presence and absence of neurotrophic factors. **A.** Representative images of RGCs treated with vehicle in the presence or absence of neurotrophic factors for 48 h. **B.** Graph bars represent total (Hoechst 33342) RGC counts, using the Mann-Whitney test. Data represent the mean ± standard error of the mean (SEM) (n = 3 biological replicates). *** $p < 0.001$. Scale bar represents 200 μm.

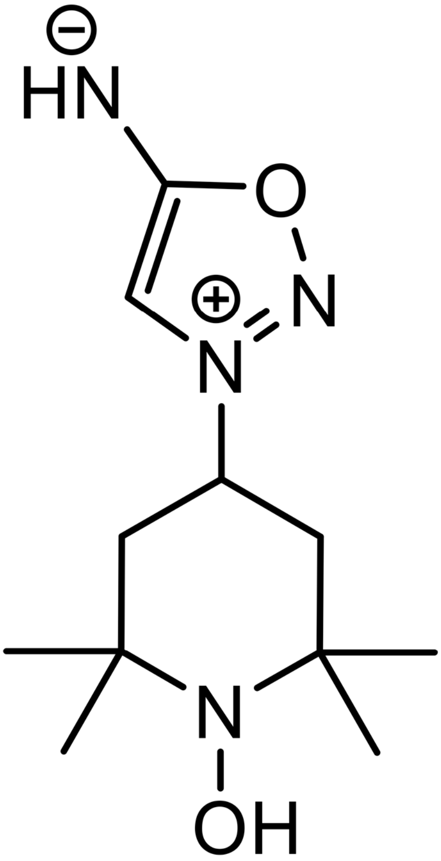

SA-2

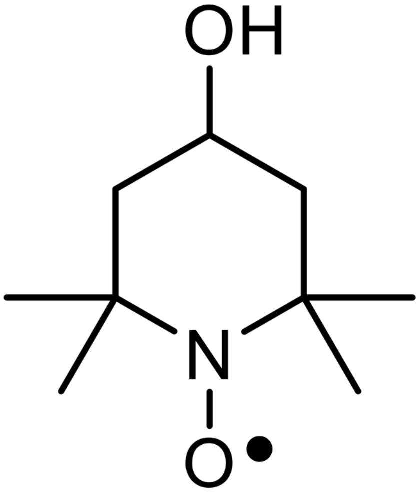

4-Hydroxy TEMPOL

Figure S2: The structure of SA-2 and 4-hydroxy TEMPOL.
